# Efficacy of Biopolymer/Starch Based Antimicrobial Packaging for Chicken Breast Fillets

**DOI:** 10.3390/foods10102379

**Published:** 2021-10-08

**Authors:** Noor L. Yusof, Noor-Azira Abdul Mutalib, U. K. Nazatul, A. H. Nadrah, Nurain Aziman, Hassan Fouad, Mohammad Jawaid, Asgar Ali, Lau Kia Kian, Mohini Sain

**Affiliations:** 1 Faculty of Food Science and Technology, Universiti Putra Malaysia, Serdang 43400, Selangor, Malaysia; noorliyana@upm.edu.my (N.L.Y.); n_azira@upm.edu.my (N.-A.A.M.); nazatulumira.karim@gmail.com (U.K.N.); nadrahabdhalid@gmail.com (A.H.N.); 2 Alliance of Research & Innovation for Food (ARIF), Faculty of Applied Sciences, Universiti Teknologi MARA, Cawangan Negeri Sembilan, Kampus Kuala Pilah, Kuala Pilah 72000, Negeri Sembilan, Malaysia; ainaziman@uitm.edu.my; 3 Applied Medical Science Department, Community College, King Saud University, P.O. Box 10219, Riyadh 11433, Saudi Arabia; menhfef@ksu.edu.sa; 4 Laboratory of Biocomposite Technology, Institute of Tropical Forestry and Forest Products (INTROP), Universiti Putra Malaysia, Serdang 43400, Selangor, Malaysia; laukiakian@gmail.com; 5 Centre of Excellence for Postharvest Biotechnology, School of Biosciences, University of Notthingham Malaysia, Semenyih 43500, Selangor, Malaysia; asgar.ali@nottingham.edu.my; 6 Centre for Biocomposites and Biomaterials Processing, University of Toronto, 33 Willcocks Street, Toronto, ON M5S3B3, Canada; m.sain@utoronto.ca

**Keywords:** biomaster-silver, SANAFOR^®^, tapioca starch, polybutylene succinate, antimicrobial, food packaging

## Abstract

Food contamination leading to the spoilage and growth of undesirable bacteria, which can occur at any stage along the food chain, is a significant problem in the food industry. In the present work, biopolymer polybutylene succinate (PBS) and polybutylene succinate/tapioca starch (PBS/TPS) films incorporating Biomaster-silver (BM) and SANAFOR^®^ (SAN) were prepared and tested as food packaging to improve the lifespan of fresh chicken breast fillets when kept in a chiller for seven days. The incorporation of BM and SAN into both films demonstrated antimicrobial activity and could prolong the storability of chicken breast fillets until day 7. However, PBS + SAN 2%, PBS/TPS + SAN 1%, and PBS/TPS + SAN 2% films showed the lowest microbial log growth. In quality assessment, incorporation of BM and SAN into both film types enhanced the quality of the chicken breast fillets. However, PBS + SAN 1% film showed the most notable enhancement of chicken breast fillet quality, as it minimized color variation, slowed pH increment, decreased weight loss, and decelerated the hardening process of the chicken breast fillets. Therefore, we suggest that the PBS + SAN and PBS/TPS + SAN films produced in this work have potential use as antimicrobial packaging in the future.

## 1. Introduction

Over the years, the food industry has faced huge food waste, especially with perishable foods. The whole process of food contamination leads to food spoilage, consequently leading to limited shelf life and low product quality. This can be seen through the changes in texture, color, and nutritive value, as well as microbial growth [1]. The storability of chicken fillets is often short term owing to their susceptibility to microbial spoilage [2]. High protein and moisture contents in the meat serve as a competent substrate for microbial growth. With the presence of oxygen, the lipid content of the chicken fillet enhances the oxidation reaction, further deteriorating the quality of the meat [3]. Thus, it depicts a higher risk for human consumption and economic loss for producers [4].

To prolong the lifespan of chicken-based foodstuffs, manufacturers have opted to wrap and package the fresh product using different types of packaging, mainly biopolymer. Biopolymer is used as an alternative to plastic packaging in producing environmentally friendly packaging. Besides reducing the accumulation of plastic waste, the use of biopolymers can also lessen the utilization of industrial agro- and biomass waste-derived plastics [5]. Polybutylene succinate (PBS) is a polymer with biocompatible characteristics that can be degraded by bacteria and fungi in landfills or the sea [6]. The mechanical and physical properties of PBS are comparable to polypropylene (PP) and polyethylene (PE), which are widely applied by the food packaging industry [7]. PBS also has the potential to replace polyolefin in the future [8]. Starch has also been considered as a polymer with biodegradable criteria besides its abundance and accessibility at a low cost. These advantages have given it great potential for packaging purposes in various fields. One of the most analyzed starch-based polymers is thermoplastic starch, which is widely applied in food packaging owing to its simple processing by common equipment in the plastic manufacturing industries, such as casting, extrusion, blow film extrusion, injection molding, and compression molding [9]. Moreover, pre-plasticized starch could promote larger surface reaction and improve homogenization during compounding thermoplastic starch/PBS thin films [10]. The addition of maleated PBS as compatibilizer to thermoplastic starch/PBS blends could provide the combined properties of great water resistance, enhanced strength, and good biodegradability, and is anticipated to serve as promising packing material [11]. However, the application of PBS is restricted due to several limitations, such as microbiological corrosion and low mechanical strength [12]. Besides that, some of the disadvantages that limit the wide use of thermoplastic starch in packaging are attributed to its self-deteriorating behavior over time as well as its high sensitivity to moisture, resulting in starch retrogradation [8]. Hence, a lot of research on different antimicrobial additives and natural fillers has been done to widen the functionality of starch-based materials [5] and PBS films.

Active packaging is a cutting-edge idea in which the package, the product, and the environment work together to extend the shelf life of foodstuffs while also improving sensory and safety characteristics, thereby retaining product quality. Antimicrobial food packaging is a type of active packaging that incorporates antimicrobial substances in food packaging to reduce pathogenic microorganisms and eliminate unpleasant changes in food quality, which in turn enhances the product’s shelf life [1,13]. Several studies on the effectiveness of antimicrobial packaging on food products have been done by previous researchers. Zhao et al. [14] produced soy protein isolation/antimicrobial silver nanoparticle films showing antibacterial activity that was efficient against Gram-positive and Gram-negative bacteria. After 28 days in a 4–10 °C temperature range, the population of Listeria monocytogenes on turkey frankfurters coated with soy protein film containing antibacterial nisin blended with either green tea extract (1%) or grape seed extract (1%) had fallen by more than 2 log cycles [15]. The bacterial flora in milk and *Micrococcus luteus* ATCC 10,240 were both effectively inhibited by the nisin-coated films [16]. Incorporation of grape seed extract also largely improved the puncture, thickness, and tensile strengths of a soy protein isolate/nisin/ethylenediaminetetraacetic acid film, and it also demonstrated the greatest inhibitory activity against *Escherichia coli* O157:H7, *Listeria monocytogenes*, and *Salmonella typhimurium* populations [17]. Another reported study fabricated ethylene–vinyl alcohol copolymer novel films mixed with cocoa extract in 10%, 15%, and 20% amounts, which presented great bactericidal activity against *Salmonella enterica*, *Escherichia coli*, *Listeria monocytogenes*, and *Staphylococcus aureus* [18]. In addition, some studies reported that the constituents of plant-derived essential oils such as lemongrass oil, oregano oil, cinnamon oil, carvacrol, cinnamaldehyde, and citral could be utilized to prepare edible-based antimicrobial films for packaging food products [19].

Recently, the development of novel antimicrobial agents against bacteria through advanced technology has been regarded as a prioritized area in the field of biomedical research. Biomaster silver (BM) is a silver-based antimicrobial material on the market. BM is based on silver ion technology, and Llorens et al. [13] mentioned that silver ions are known to possess the highest antimicrobial capacity of the metallic cations and is able to inhibit a wide range of microbial organisms. It possesses low vitality and long-term biocide properties, yet a low level of toxicity to eukaryotic cells. The interaction of silver ions with the ribosomes would inhibit the enzyme expression and interfere with the permeability of the membrane. It interacts with nucleic acids and cytoplasmatic components, and alters the enzyme mechanism behavior after binding with protein thiol groups [20]. According to Xu et al. [21], previous studies have shown that an increase in reactive oxygen species (ROS) was induced by the presence of metal nanoparticles, posing toxic effects correlated with oxidative stress. The inhibition of respiratory enzymes was caused by silver ions that interacted with the thiol group of the enzymes, thus producing ROS. It is considered one of the critical factors that can affect oxidative stress, as ROS is able to react directly with DNA and proteins or with lipids in order to induce the production of malondialdehyde, which can react with DNA, proteins, and other lipids [22]. A higher temperature has also been proven to induce higher antibacterial activity in silver nanoparticles through the increase of the ROS formation level [21]. Apart from that, SANAFOR^®^ (SAN), a commercialized antimicrobial agent, controls microbial growth on plastic surfaces, protecting against bacteria, fungi, and algae that may degrade the food products. Although the active substances used in the antimicrobial agent were not mentioned, it was said that SAN could provide good thermal stability with low water solubility that may limit the migration from plastic.

The goal of this work is to address the various modifications and preparation methodologies of preparing BM and SAN as promising materials that can be applies as coating or packaging material in food, providing new insights into the numerous applications to maintain the quality and safety of the food products contained in the packaging for a specific time period. Although BM and SAN are not newly developed antimicrobial agents, the incorporation of both compounds has not been fully explored when it comes to its application in food packaging, especially for a perishable commodity like poultry. There are no available studies focusing on incorporating polybutylene succinate (PBS) and polybutylene succinate/tapioca starch (PBS/TPS) with BM and SAN per se, and these materials exhibited strong antimicrobial activity against microorganisms, especially in poultry products. Hence, the idea of incorporating BM and SAN into PBS/TPS film could open up new possibilities of developing a sustainable packaging film for preserving a few other perishable foods. The novelty of this study is that the modification of BM and SAN with different contents was done to tailor the optimum concentration that can be useful in food packaging, especially to ensure the microbiological safety and maintain the physicochemical quality of chicken breast fillets during storage, as well as its environmentally friendly materials. Antimicrobial packaging, on the other hand, is still a difficult technology to master, with only a few items commercialized on the market. Furthermore, based on our knowledge, no study has been conducted regarding the incorporation of BM and SAN into PBS and PBS/TPS films. Therefore, the present work aims to investigate the effects of PBS and PBS/TPS films incorporated with BM and SAN on the shelf life of fresh chicken breast fillets. Therefore, the novelty of this study is the generation of antimicrobial packaging films from PBS and PBS/TPS materials for food preservation purposes.

## 2. Materials and Methods

### 2.1. Materials

PBS pellets (BioPBS™ FZ71PB) (density: 1.26 g/cm^3^) were purchased from PTT Public Company Limited in Bangkok, Thailand. The powder form of tapioca starch (TPS) was bought from PT Starch Solution in Karawang, Indonesia. Meanwhile, Biomaster-silver (BM) and SANAFOR^®^ (SAN) were purchased from Malaysian companies. Other materials like agar plate and peptone water were procured from Oxoid, Malaysia. Fresh chicken breast fillets were obtained from a supply chain company, Segi Fresh, Balakong, Selangor.

### 2.2. Fabrication of PBS and PBS/TPS Films

In this study, PBS and PBS/TPS films were prepared through the melting-blow method since this technique can facilitate the dispersion of different added components with the PBS matrix. Two distinct antimicrobial substances, namely, BM and SAN, were incorporated into two different films, (i) PBS and (ii) PBS, with the addition of TPS. The antimicrobial substances were incorporated at different concentrations. Two untreated films, PBS film and PBS film, were prepared with TPS (PBS/TPS) added. A total of 10 films were provided, as shown in Table 1. The films were manually laminated onto a kraft paper tray (11.5 × 9 × 5 cm) aseptically. As for the control, a polypropylene (PP) microwavable container was used.

### 2.3. Sampling and Storage

The chicken breast fillet samples, weighing approximately 30 ± 3 g, were placed on the laminated kraft paper tray. The trays were covered with the same lamination film, then kept for 7 days at 4 °C. The procedure was carried out in triplicate for each sample. Microbiological analysis and quality assessment were conducted on days 0, 1, 3, 5, and 7 during storage. The analyses on the initial day (day 0) were carried out upon freshly bought chicken fillets with no treatment applied.

### 2.4. Microbiological Analysis

Microbiological analysis was determined by total plate count (TPC) according to CLSI (2010). The chicken breast fillets were cut into smaller pieces with a weight of around 25 g. Each sample was then moved to stomacher bags that were filled with 225 mL of 0.1% sterilized peptone solution, followed by 60 s homogenization within the stomacher (Tekma Lab Blender 80, Seward Medical, UK). Suitable serial dilution was then applied to the peptone water (0.1%) contained in each sample. Afterwards, about 0.1 mL of the diluted homogenates were evenly distributed on Plate Count Agar (Oxoid, UK) using an L-shaped rod. Then, the inoculated plates underwent incubation for 18–24 h at 37 °C. The TPC was counted for each sample as log CFU/g, representing the logarithms of colony-forming units per gram [4].

### 2.5. Quality Assessment

#### 2.5.1. pH Analysis

The pH values of the chicken samples were examined following the AOAC method. Approximately 10 g sample were mixed with 100 mL distilled water and then the filtrate was collected for pH measurement using a pH meter (Mettler-Toledo International Inc., Columbus, OH, USA).

#### 2.5.2. Weight Loss

The changes in the weight of the chicken breast fillet samples were recorded on days 1, 3, 5, and 7 upon storage. The weight loss was presented as lost percentage in respect to the initial weight of the samples [4].

#### 2.5.3. Texture Analysis

The texture of the chicken breast fillet samples was analyzed with a texture analyzer (TA.HDplusC, Stable Micro Systems Ltd., Godalming, UK), and the texture was described in terms of hardness. Three different positions were located for each sample with perpendicular measurements to the chicken breast fillet surface, whereas the mean values were analyzed in triplicate measurement from the fillet samples to obtain an average value of the hardness [23].

#### 2.5.4. Color Analysis

The measurement of color for the chicken breast fillets was performed with a chroma meter (CR-410, Konica Minolta, Japan). Values of a* (redness), b* (yellowness), and L* (lightness) were used to characterize color. Three different positions were located for each sample with perpendicular measurements to the chicken breast fillet surface [24]. The mean values (a*, b*, and L*) were analyzed in triplicate measurement from the fillet samples to obtain an average value of the hardness [25].

#### 2.5.5. Overall Visual Quality

The overall observation of the chicken breast fillet samples throughout the storage was conducted with a camera (Apple iPhone 6 Plus, Apple Inc., Cupertino, CA, USA).

### 2.6. Statistical Analysis

Triplicate work was conducted for all analyses in this study, and the collected data were analyzed by one-way ANOVA to obtain the values of mean ± standard deviation. Meanwhile, the statistical differences were regarded as significant as if *p* < 0.05 (Duncan’s multiple range test). All statistical evaluations for the results were performed using Minitab 18.0 Statistical software.

## 3. Results

### 3.1. Total Plate Count (TPC)

**Figure 1:**
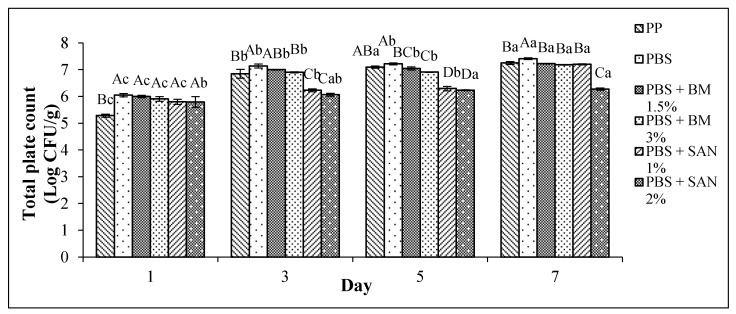
Total plate count (TPC) of chicken breast fillets stored in PP container and PBS film laminated tray during storage at 4 °C for seven days. Note: PP: polypropylene microwavable container (control); PBS: PBS film (untreated); PBS + BM 1.5%: PBS film + 1.5% Biomaster silver; PBS + BM 3%: PBS film + 3% Biomaster silver; PBS + SAN 1%: PBS film + 1% SANAFOR; PBS + SAN 2%: PBS film + 2% SANAFOR. Error bars indicate standard deviation (n = 3). The different A–D capital letters are significantly different (*p* < 0.05) among treatment for each day. The different a–c lowercase letters are significantly different (*p* < 0.05) among storage day for each sample.

**Figure 2:**
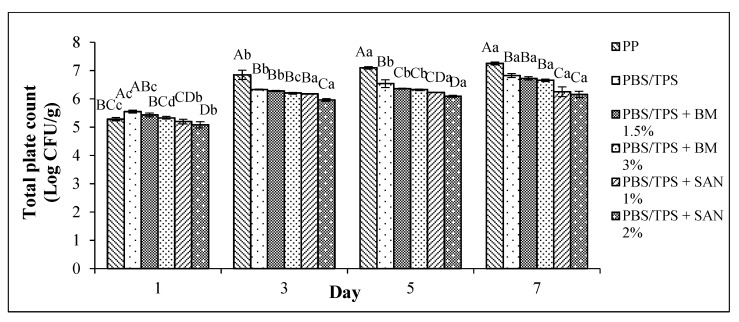
Total plate count (TPC) of chicken breast fillets stored in PP container and PBS/TPS film laminated tray during storage at 4 °C for seven days. Note: PP: polypropylene microwavable container (control); PBS/TPS: PBS/TPS film (untreated); ¯PBS/TPS + BM 1.5%: PBS/TPS film + 1.5% Biomaster silver; PBS/TPS + BM 3%: PBS/TPS film + 3% Biomaster silver; PBS/TPS + SAN 1%: PBS/TPS film + 1% SANAFOR; PBS/TPS + SAN 2%: PBS/TPS film + 2% SANAFOR. Error bars indicate standard deviation (n = 3). The different A–D capital letters are significantly different (*p* < 0.05) among treatment for each day. The different a–d lowercase letters are significantly different (*p* < 0.05) among storage day for each sample.

The TPC of the chicken breast fillet samples stored in the PP container and PBS film laminated tray during storage at 4 °C for seven days is shown in Figure 1. Meanwhile, the TPC of the chicken breast fillet samples stored in the PP container and PBS/TPS film laminated tray during storage at 4 °C for seven days is shown in Figure 2.

The initial TPC of the chicken samples without any treatment (day 0) was 4.17 log CFU/g. Based on Figure 1, the log CFU/g value increased in all treatments during storage at 4 °C up to seven days, with values between 4.17 and 7.41 log CFU/g. The limit of acceptability for meat product is 7.00 log CFU/g [26]. Among all samples, the untreated PBS and PBS + BM 1.5% exceeded the acceptability limit on the third day of storage. However, the PP container (control) surpassed the acceptability limit on the fifth day. The untreated PBS and PBS + BM 1.5% films showed higher TPC than the control throughout the seven days, as PBS showed low gas barrier properties [27] that may induce microbial growth. The incorporation of 3% BM and 1% SAN into the PBS improved the film antimicrobial activity, and the lifespan of chicken samples stored in PBS + BM 3% and PBS + SAN 1% films could be extended up to seven days. Meanwhile, the chicken sample stored in the PBS + SAN 2% film showed the significantly lowest (*p* < 0.05) TPC and was still acceptable on day 7, indicating that 2% SAN exhibits the highest antimicrobial properties compared to the others.

As illustrated in Figure 2, the log CFU/g value increased in all treatments during storage at 4 °C up to seven days with values between 4.17 and7.25 log CFU/g. The addition of TPS on both untreated and treated films resulted in lower log values than the control from day 3 storage onwards. This further indicates that the addition of TPS to PBS made it partially compatible polymer blends [11]. Among all the samples, the PP container (control) exceeded the acceptability limit on the fifth day of storage; however, other treatments for chicken samples were still acceptable up to seven days. Among the treated films, the incorporation of 1% and 2% SAN into PBS/TPS enhanced the antimicrobial activity by exhibiting the significantly lowest (*p* < 0.05) log value compared to the others up to seven days.

Appendini and Hotchkiss [28] stated that the introduction of antimicrobials into the packaging is to avoid surface growth on foods, such as the spoilage that may occur primarily on the surface of intact meat. The antimicrobials were gradually released from the packaging film, which may be better compared to the dipping and spraying technique. The techniques of directly adding preservatives may cause rapid diffusion of the antibacterial agent into food and denaturize its active sites, which ultimately lowers the reactivity with bacteria. However, antimicrobial-incorporated packaging allows antimicrobial agents to migrate slowly and continuously from the container to the food surface, which helps enhance the high concentration of antimicrobial agents over a longer period [2]. They also discovered that as the storage temperature rises, the movement of active chemicals from film to food accelerates. We did not observe this effect in the present study since our chicken samples were stored at 4 °C, and the interactions between coating materials, target bacteria, antimicrobial agents, and the food matrix themselves could differ, therefore influencing active compound release rates.

As reported by Warsiki and Bawardi [29], antibacterial packaging materials could be made from tapioca starch films containing antimicrobial ZnO nanoparticles. After integration with 2% ZnO, the film showed an inhibition index of around 7.67 mm against E. coli. PBS with 10 wt.% thymol was also effective for inhibiting E. coli growth [12]. Cardoso et al. [30] tested the antimicrobial efficiency of oregano essential oil (OEO)-filled poly (butylene adipate co-terephthalate) active films for fish fillet preservation at 7 °C. All formulations except 2.5 g OEO showed improved fillet shelf life reaching up to 10 days. The polylactic acid-based composite films containing 50% cinnamon oil presented antimicrobial behavior against Salmonella Typhimurium and Listeria monocytogenes when inoculated in chicken samples for 16 days of storage in refrigerated conditions [31]. Chitosan films incorporated with 2% ethanolic propolis extract and 1–2% cellulose nanoparticle was also a viable option in slowing microbial development as well as protein and lipid oxidation in minced beef meat [32].

In this study, the antimicrobial agents BM and SAN were effective in PBS and PBS/TPS films. The antimicrobial mechanism of BM silver ion technology is based on the release of silver ions into the moisture layer that naturally exists on a product’s surface, then permeates through bacterial cell walls, deactivating essential energy-producing metabolic enzymes and stopping bacteria from growing [33]. However, the mechanism by which SAN inhibits bacteria growth has not been elucidated.

Overall, the log value of the PBS/TPS films with antimicrobial agents was lower than in the PBS films with antimicrobial agents. Furthermore, SAN was shown to have lower microbial growth and was more effective than BM in both PBS and PBS/TPS films. This study concluded that PBS with 2% SAN and PBS/TPS with 1% SAN or 2% SAN were more effective in serving as antimicrobial packaging.

### 3.2. pH Value

**Figure 3:**
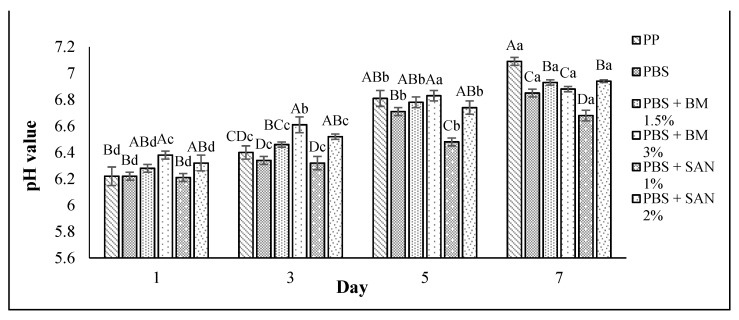
The pH values of chicken breast fillets stored in PP container and PBS film laminated tray during storage at 4 °C for seven days. Note: PP: polypropylene microwavable container (control); PBS: PBS film (untreated); PBS + BM 1.5%: PBS film + 1.5% Biomaster silver; PBS + BM 3%: PBS film + 3% Biomaster silver; PBS + SAN 1%: PBS film + 1% SANAFOR; PBS + SAN 2%: PBS film + 2% SANAFOR. Error bars indicate standard deviation (n = 3). The different A–D capital letters are significantly different (*p* < 0.05) among treatment for each day. The different a–d lowercase letters are significantly different (*p* < 0.05) among storage day for each sample.

**Figure 4:**
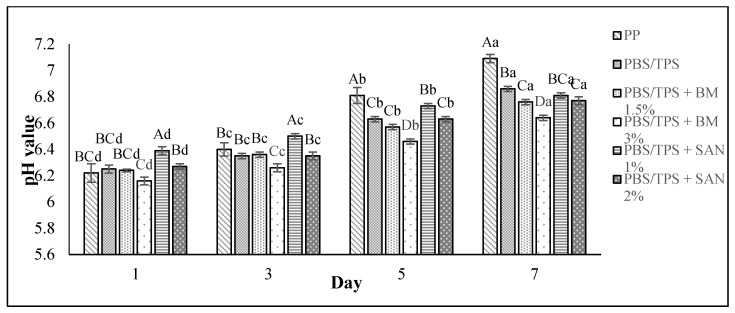
The pH values of chicken breast fillets stored in PP container and PBS/TPS film laminated tray during storage at 4 °C for seven days. Note: PP: polypropylene microwavable container (control); PBS/TPS: PBS/TPS film (untreated); PBS/TPS + BM 1.5%: PBS/TPS film + 1.5% Biomaster silver; PBS/TPS + BM 3%: PBS/TPS film + 3% Biomaster silver; PBS/TPS + SAN 1%: PBS/TPS film + 1% SANAFOR; PBS/TPS + SAN 2%: PBS/TPS film + 2% SANAFOR. Error bars indicate standard deviation (n = 3). The different A–D capital letters are significantly different (*p* < 0.05) among treatment for each day. The different a–d lowercase letters are significantly different (*p* < 0.05) among storage day for each sample.

The pH changes in value for the chicken samples stored in PBS and PBS/TPS film laminated tray during storage at 4 °C for seven days are shown in Figure 3 and Figure 4, respectively. The pH value shown by the chicken samples initially in this study without any treatment (day 0) was 6.15 ± 0.06.

Our findings show that as the storage period increased, the pH value of the chicken samples increased. However, the pH values of the chicken samples in PBS and PBS/TPS films incorporating BM and SAN were slightly increased compared to the control sample. The chicken samples stored in PP container (control) showed the highest pH value (7.09 ± 0.03) at the end of storage, and a pH value above 7 is considered to indicate negative sensory attributes [34].

Similar trends of pH increment throughout the storage period of chicken and beef were also observed in recent studies by Rashidaie et al. [35] and Katiyo et al. [36]. The accumulated alkali nitrogenous components such as amines and ammonia produced by either microbial or endogenous enzymes could cause an increased pH value, which subsequently leads to the meat color darkening [36,37]. Therefore, the low pH increment of chicken samples stored in the treated films in this study might be contributed to the low color variation of chicken samples, as discussed earlier in this study. The slow pH increment in chicken samples stored in treated films might be due to the ability of antimicrobial agents BM and SAN to prohibit enzyme activity [37] and low oxidation rates occurred during storage [34]. When compared to unpackaged poultry, samples packed with chitosan/montmorillonite bionanocomposites incorporating ginger essential oil showed a 1.2 to 2.6 log CFU/g reduction in microbial count and were able to maintain their color and pH values [38]. Therefore, the pH values of chicken samples were correlated with the total plate count and color. Overall, the lowest pH values were obtained for the chicken breast fillets samples stored in PBS + SAN 1% and PBS/TPS + BM 3% films.

### 3.3. Weight Loss

**Figure 5:**
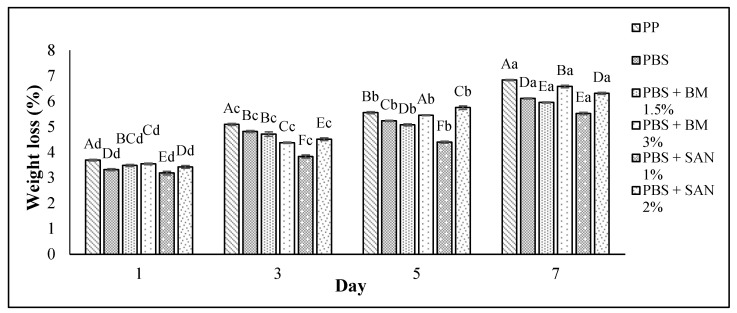
The weight loss values of chicken breast fillets stored in PP container and PBS film laminated tray during storage at 4 °C for seven days. Note: PP: polypropylene microwavable container (control); PBS: PBS film (untreated); PBS + BM 1.5%: PBS film + 1.5% Biomaster silver; PBS + BM 3%: PBS film + 3% Biomaster silver; PBS + SAN 1%: PBS film + 1% SANAFOR; PBS + SAN 2%: PBS film + 2% SANAFOR. Error bars indicate standard deviation (n = 3). The different A–F capital letters are significantly different (*p* < 0.05) among treatment for each day. The different a–d lowercase letters are significantly different (*p* < 0.05) among storage day for each sample.

**Figure 6:**
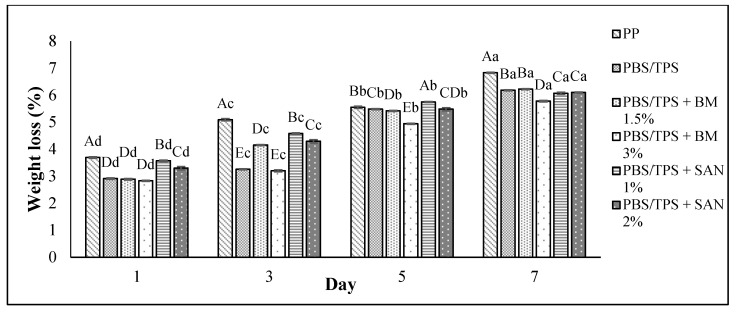
The weight loss values of chicken breast fillets stored in PP container and PBS/TPS film laminated tray during storage at 4 °C for seven days. Note: PP: polypropylene microwavable container (control); PBS/TPS: PBS/TPS film (untreated); PBS/TPS + BM 1.5%: PBS/TPS film + 1.5% Biomaster silver; PBS/TPS + BM 3%: PBS/TPS film + 3% Biomaster silver; PBS/TPS + SAN 1%: PBS/TPS film + 1% SANAFOR; PBS/TPS + SAN 2%: PBS/TPS film + 2% SANAFOR. Error bars indicate standard deviation (n = 3). The different A–E capital letters are significantly different (*p* < 0.05) among treatment for each day. The different a–d lowercase letters are significantly different (*p* < 0.05) among storage day for each sample.

The weight loss of the chicken breast fillet samples stored in the PBS and PBS/TPS film laminated tray during storage at 4 °C for seven days is shown in Figure 5 and Figure 6, respectively. Based on both figures, the weight loss increased (*p* < 0.05) throughout seven days of the storage period for all samples, with values between 3.18 and 6.83% (Figure 5) and 2.83 and 6.83% (Figure 6).

The weight loss of the chicken samples in PBS and PBS/TPS film incorporating BM and SAN exhibited a slight increase throughout the period of storage compared to the control sample. The results show that chicken samples stored in PBS + SAN 1% and PBS + BM 1.5% film (Figure 5) and PBS/TPS + BM 3% film (Figure 6) recorded the lowest weight loss on day 7 (5.52 ± 0.04%, 5.95 ± 0.02%, and 5.78 ± 0.03%, respectively) compared to the chicken samples stored in the PP container (control) and other films. The increase in weight loss throughout storage was due to the moisture loss from the chicken breast fillets and further resulted in the hardening of the meat texture [34]. As reported by Amjadi et al. [39], the chicken fillets wrapped with ZnO nanoparticles and chitosan nanofiber-filled gelatin-based nanocomposite exhibited less weight loss than the control at the end of the 12th day of storage.

### 3.4. Texture Analysis

**Figure 7:**
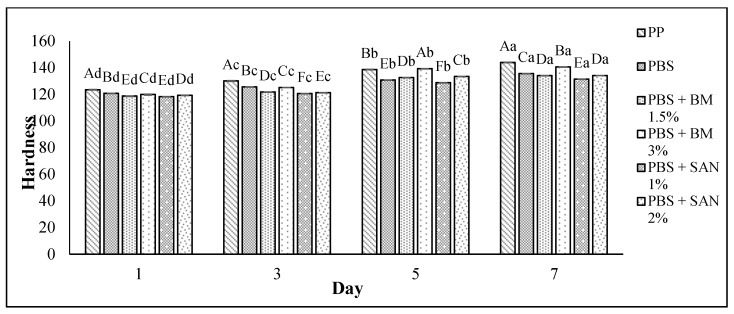
The hardness values of chicken breast fillets stored in PP container and PBS film laminated tray during storage at 4 °C for seven days. Note: PP: polypropylene microwavable container (control); PBS: PBS film (untreated); PBS + BM 1.5%: PBS film + 1.5% Biomaster silver; PBS + BM 3%: PBS film + 3% Biomaster silver; PBS + SAN 1%: PBS film + 1% SANAFOR; PBS + SAN 2%: PBS film + 2% SANAFOR. Error bars indicate standard deviation (n = 3). The different A–F capital letters are significantly different (*p* < 0.05) among treatment for each day. The different a–d lowercase letters are significantly different (*p* < 0.05) among storage day for each sample.

**Figure 8:**
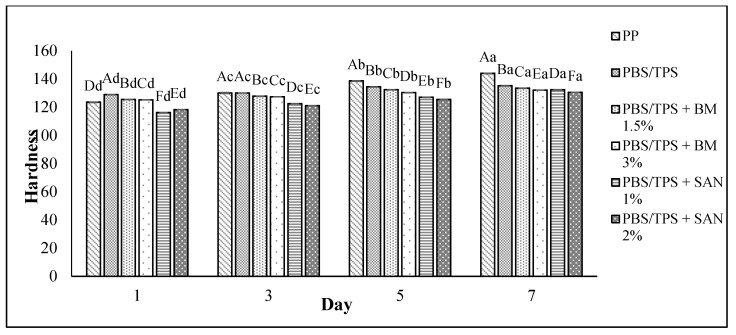
The hardness values of chicken breast fillets stored in PP container and PBS/TPS film laminated tray during storage at 4 °C for seven days. Note: PP: polypropylene microwavable container (control); PBS/TPS: PBS/TPS film (untreated); PBS/TPS + BM 1.5%: PBS/TPS film + 1.5% Biomaster silver; PBS/TPS + BM 3%: PBS/TPS film + 3% Biomaster silver; PBS/TPS + SAN 1%: PBS/TPS film + 1% SANAFOR; PBS/TPS + SAN 2%: PBS/TPS film + 2% SANAFOR. Error bars indicate standard deviation (n = 3). The different A–F capital letters are significantly different (*p* < 0.05) among treatment for each day. The different a–d lowercase letters are significantly different (*p* < 0.05) among storage day for each sample.

The texture of the chicken breast fillet samples was analyzed in terms of hardness. The hardness values of the chicken breast fillet samples stored in the PBS and PBS/TPS film laminated tray during storage at 4 °C for seven days are shown in Figure 7 and Figure 8, respectively. The hardness values increased (*p* < 0.05) throughout the seven days of the storage period for all samples, with values between 118.31 and 144.02% (Figure 7) and 116.22 and 144.02% (Figure 8). Compared to the control sample, the chicken samples stored in PBS and PBS/TPS film exhibited less hardness.

The lowest hardness of chicken fillets stored in the treated films might have been due to their lower weight loss, as discussed in Section 3.4, indicating that the treated films decreased the loss of weight, and then enhanced the texture of the chicken samples during storage at 4 °C. Based on Figure 7, the results show that the chicken samples stored in PBS + SAN 1% film recorded the lowest hardness value (131.48 ± 0.04) at the end of storage. Meanwhile, Figure 8 shows that the chicken samples stored in PBS/TPS + SAN 2% film had the lowest hardness value (130.57 ± 0.03).

### 3.5. Color Changes and Overall Visual Quality

**Figure 9:**
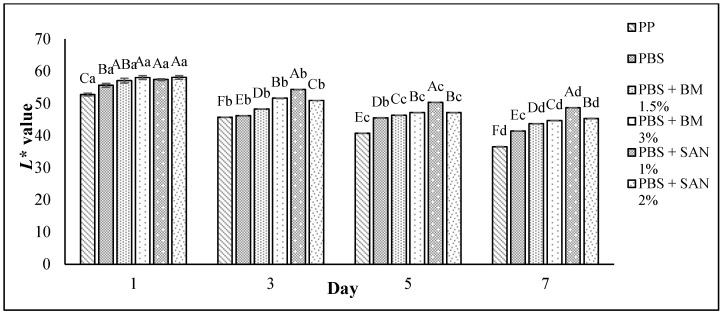

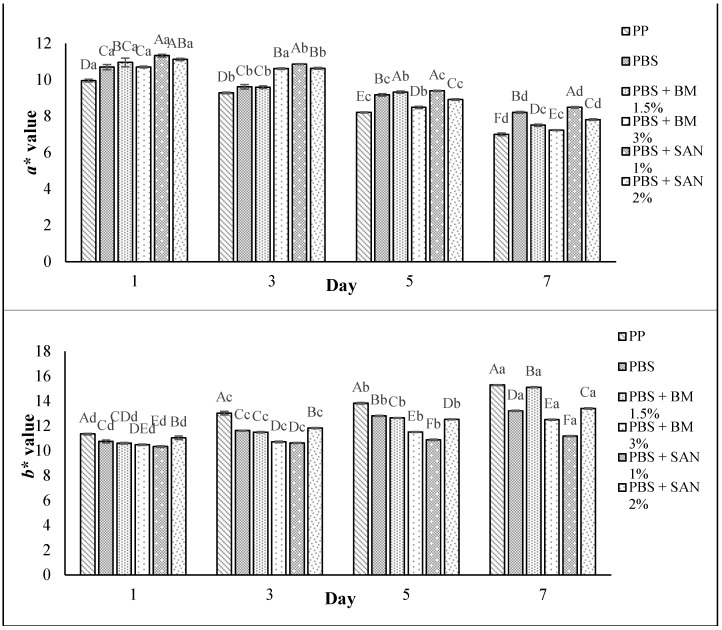
The L*, a*, and b* values of chicken breast fillets stored in PP container and PBS film laminated tray during storage at 4 °C for seven days. Note: Color parameters: L* (from 0 black to 100 white), a* (from -a* green to +a* red), and b* (from -b* blue to +b* yellow). PP: polypropylene microwavable container (control); PBS: PBS film (untreated); PBS + BM 1.5%: PBS film + 1.5% Biomaster silver; PBS + BM 3%: PBS film + 3% Biomaster silver; PBS + SAN 1%: PBS film + 1% SANAFOR; PBS + SAN 2%: PBS film + 2% SANAFOR. Error bars indicate standard deviation (n = 3). The different A–F capital letters are significantly different (*p* < 0.05) among treatment for each day. The different a–d lowercase letters are significantly different (*p* < 0.05) among storage day for each sample.

In contemporary packing technologies, one of the primary assumptions is that the desired color would be preserved for as long as possible [40]. The overall visual quality of the chicken breast fillet samples stored in the PP container and PBS film laminated tray during storage at 4 °C for seven days is shown in Table 2. Table 2 shows that chicken samples stored in a tray laminated with PBS + SAN 1% film had the most pleasant appearance compared to the chicken samples stored in the PP container and other films. The chicken sample was still fresh on day 3 of storage and the color variation was not significant as compared to the other chicken samples, and no bad odor was detected once the lid was opened. The color (L*, a*, and b* values) of the chicken fillet samples kept in the PP container and PBS film laminated tray during storage at 4 °C for seven days is shown in Figure 9. In this study, the initial L*, a*, and b* values of the chicken fillets without any treatment (day 0) were 56.02 ± 0.21, 12.01 ± 0.08, and 10.29 ± 0.12, respectively.

According to Figure 9, the L* (lightness) and a* (redness) values for each chicken sample decreased (*p* < 0.05) gradually throughout the seven-day storage period storage. However, the chicken fillets presented increased (*p* < 0.05) b* (yellowness) values over the period of storage. The L* values were between 36.50 and 58.06, the a* values were between 7.00 and 11.33, and the b* values were between 10.33 and 15.29. The decrease in L* and a* values for all chicken fillets throughout the storage period of seven days indicates that the chicken darkened throughout the storage. The L* value of chicken samples stored in PBS film incorporated with BM and SAN was able to slow down the chicken darkening during the seven-day storage period compared to the PP container (control) and untreated PBS. The PBS + SAN 1% film exhibited the highest (*p* < 0.05) L* value, followed by PBS + SAN 2%, PBS + BM 3%, and PBS + BM 1.5% films. The PBS + SAN 1% also showed the highest (*p* < 0.05) a* value on day 7. The chicken samples stored in PBS + SAN 1% film recorded the lowest b* value (*p* < 0.05) compared to the chicken samples in other films.

**Figure 10:**
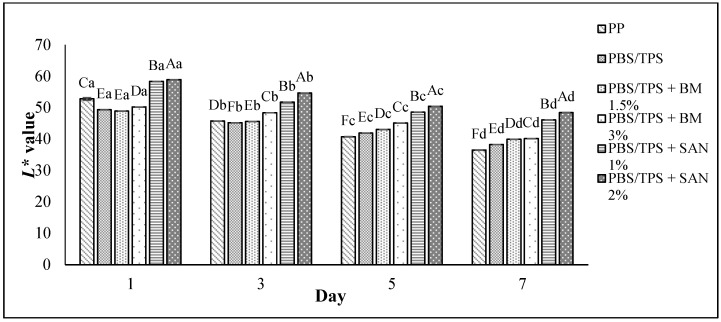

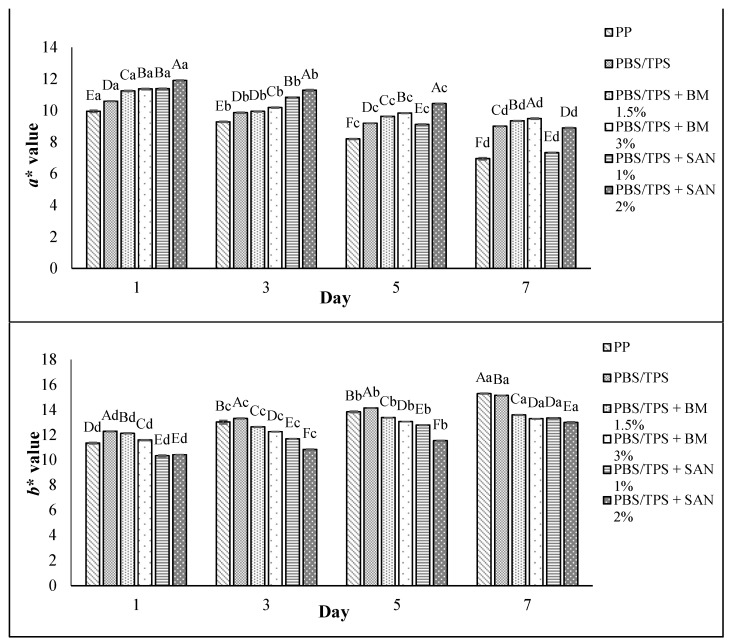
The L*, a*, and b* values of chicken breast fillets stored in PP container and PBS/TPS film laminated tray during storage at 4 °C for seven days. Note: Color parameters: L* (from 0 black to 100 white), a* (from -a* green to +a* red), and b* (from -b* blue to +b* yellow). PP: polypropylene microwavable container (control); PBS/TPS: PBS/TPS film (untreated); PBS/TPS + BM 1.5%: PBS/TPS film + 1.5% Biomaster silver; PBS/TPS + BM 3%: PBS/TPS film + 3% Biomaster silver; PBS/TPS + SAN 1%: PBS/TPS film + 1% SANAFOR; PBS/TPS + SAN 2%: PBS/TPS film + 2% SANAFOR. Error bars indicate standard deviation (n = 3). The different A–F capital letters are significantly different (*p* < 0.05) among treatment for each day. The different a–d lowercase letters are significantly different (*p* < 0.05) among storage day for each samples.

The overall visual quality of the chicken breast fillet samples stored in the PP container and PBS/TPS film laminated tray during storage at 4 °C for seven days is shown in Table 3. Based on Table 3, the chicken samples stored in PBS/TPS + SAN 1% and PBS/TPS + SAN 2% had the most appealing appearance compared to the chicken samples in the PP container and other films. The chicken fillets were still fresh on the third day of storage, and the color variation did not significantly change. The color (L*, a*, and b* values) of the chicken fillet samples kept in the PP container and PBS/TPS film laminated tray during storage at 4 °C for seven days is shown in Figure 10.

According to Figure 10, the L* (lightness) and a* (redness) values for all chicken samples decreased (*p* < 0.05) gradually throughout the seven-days of the storage period. However, the chicken samples showed increased (*p* < 0.05) b* (yellowness) values over the storage period. The L* values were between 36.50 and 58.33, the a* values were between 6.95 and 11.90, and the b* values were between 10.33 and 15.29. The L* value of the chicken samples stored in PBS/TPS films incorporated with BM and SAN were able to slow down the darkening of the chicken samples throughout the seven days of the storage period compared to the PP container (control) and untreated PBS/TPS, with PBS/TPS + SAN 2% exhibiting the highest (*p* < 0.05) L* value, followed by PBS/TPS + SAN 1%, PBS/TPS + BM 3%, PBS/TPS + BM 1.5%, PBS/TPS, and PP. However, the chicken samples stored in PBS/TPS + BM3% film showed the highest (*p* < 0.05) a* value on day 7, followed by the PBS/TPS + BM 1.5% film. The chicken sample stored in the PBS/TPS + SAN 2% film showed the lowest (*p* < 0.05) b* value on day 7, followed by PBS/TPS + SAN 1%, PBS/TPS + BM 3%, PBS/TPS + BM 1.5%, PBS/TPS, and PP.

From the color analysis, it can be concluded that chicken breast fillets stored in PBS + SAN 1% and PBS/TPS + SAN 2% showed the least significant color variation throughout the seven days of the storage period compared to the chicken breast fillets stored in the PP container and other films. Both of these films are the most efficient for slowing the darkening behavior of chicken breast fillets resulting from the accumulation of ammonia and amines and retaining the red color of the chicken breast fillets [36].

## 4. Conclusions

In conclusion, the addition of TPS to PBS film showed a lower microbial log growth than PBS film alone. With antimicrobial agents BM and SAN being added to the packaging material, it further aids in monitoring the microbial growth and enhances the storability of chicken breast fillets up to seven days. However, the incorporation of 2% SAN into PBS film and 1% or 2% SAN into PBS/TPS films showed the highest potential as an antimicrobial property in reducing the log value compared to other film packaging. The quality assessment proved that the incorporation of BM and SAN into the films can enhance the quality of chicken breast fillets by minimizing the color variation, slowing pH increment, decreasing weight loss, and decelerating the hardening process of the chicken breast fillets throughout storage. The PBS + SAN 1% and PBS/TPS + SAN 2% films exhibited the least color variation. The PBS + SAN 1% and PBS/TPS + BM 3% films showed the least pH increment. The PBS + SAN 1%, PBS + BM 1.5%, and PBS/TPS + BM 3% films showed the least weight loss. The PBS + SAN 1% and PBS/TPS + SAN 2% films exhibited the least hardness. However, the most notable enhancement of the chicken breast fillets’ quality was observed in the PBS film incorporated with 1% SAN, as it recorded the least color variation, pH increment, weight loss, and hardness. Therefore, it has potential to be used as new antimicrobial packaging material for chicken fillets and could be an alternative to plastic packaging.

## Figures and Tables

**Table 1:** A total of 10 formulations of PBS films.

No.	PBS Film Code	Antimicrobial Agent	g/g (%) Amount of Antimicrobial Agent
1	PBS (untreated)	N/A	N/A
2	PBS + BM 1.5%	BM	1.5
3	PBS + BM 3%	BM	3
4	PBS + SAN 1%	SAN	1
5	PBS + SAN 2%	SAN	2
4	PBS/TPS (untreated)	N/A	N/A
5	PBS/TPS + BM 1.5%	BM	1.5
6	PBS/TPS + BM 3%	BM	3
7	PBS/TPS + SAN 1%	SAN	1
8	PBS/TPS + SAN 2%	SAN	2

Note: N/A: not available.

**Table 2:** The overall visual quality of chicken breast fillets stored in PP container and PBS film laminated tray during storage at 4 °C for seven days.

Day	0	1	3	5	7
PP	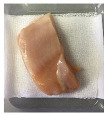	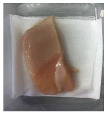	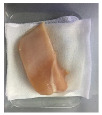	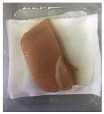	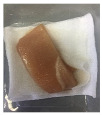
PBS	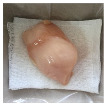	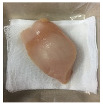	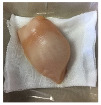	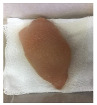	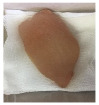
PBS + BM 1.5%	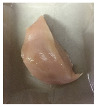	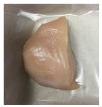	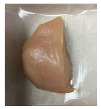	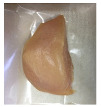	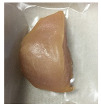
PBS + BM 3%	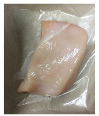	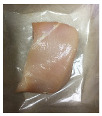	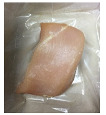	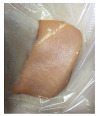	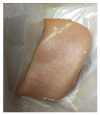
PBS + SAN 1%	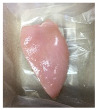	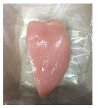	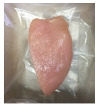	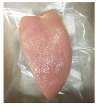	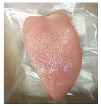
PBS + SAN 2%	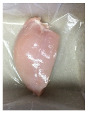	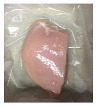	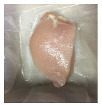	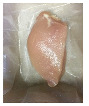	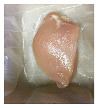

Note: PP: polypropylene microwavable container (control); PBS: PBS film (untreated); PBS + BM 1.5%: PBS film + 1.5% Biomaster silver; PBS + BM 3%: PBS film + 3% Biomaster silver; PBS + SAN 1%: PBS film + 1% SANAFOR; PBS + SAN 2%: PBS film + 2% SANAFOR.

**Table 3:** The overall visual quality of chicken breast fillets stored in PP container and PBS/TPS film laminated tray during storage at 4 °C for seven days.

Day	0	1	3	5	7
PP	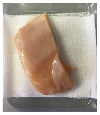	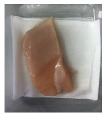	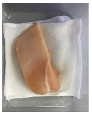	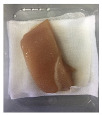	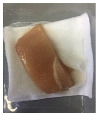
PBS/TPS	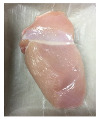	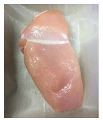	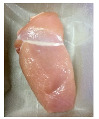	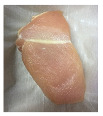	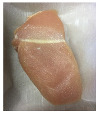
PBS/TPS + BM 1.5%	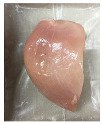	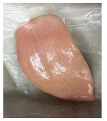	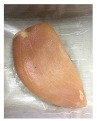	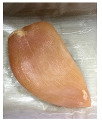	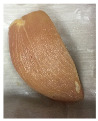
PBS/TPS + BM 3%	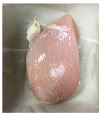	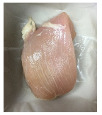	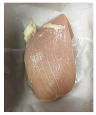	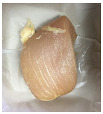	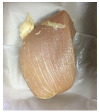
PBS/TPS + SAN 1%	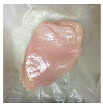	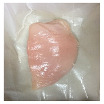	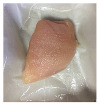	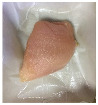	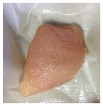
PBS/TPS + SAN 2%	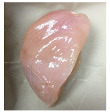	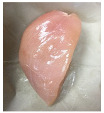	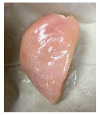	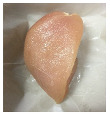	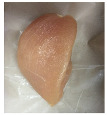

Note: PP: polypropylene microwavable container (control); PBS/TPS: PBS/TPS film (untreated); PBS/TPS + BM 1.5%: PBS/TPS film + 1.5% Biomaster silver; PBS/TPS + BM 3%: PBS/TPS film + 3% Biomaster silver; PBS/TPS + SAN 1%: PBS/TPS film + 1% SANAFOR; PBS/TPS + SAN 2%: PBS/TPS film + 2% SANAFOR.

## Data Availability

Not applicable.
